# Evaluation of Novel Large Cut-Off Ultrafiltration Membranes for Adenovirus Serotype 5 (Ad5) Concentration

**DOI:** 10.1371/journal.pone.0115802

**Published:** 2014-12-29

**Authors:** Piergiuseppe Nestola, Duarte L. Martins, Cristina Peixoto, Susanne Roederstein, Tobias Schleuss, Paula M. Alves, José P. B. Mota, Manuel J. T. Carrondo

**Affiliations:** 1 Instituto de Biologia Experimental e Tecnológica, Apartado 12, 2781-901, Oeiras, Portugal; 2 Instituto de Tecnologia Química e Biológica, Universidade Nova de Lisboa, 2780-157, Oeiras, Portugal; 3 Requimte/CQFB, Departamento de Química, Faculdade de Ciências e Tecnologia, Universidade Nova de Lisboa, 2829-516, Caparica, Portugal; 4 Departamento de Química, Faculdade de Ciências e Tecnologia, Universidade Nova de Lisboa, 2829-516, Caparica, Portugal; 5 Sartorius Stedim Biotech, Spindler-Strasse 11, 37079, Gottingen, Germany; Tecnologico de Monterrey, Mexico

## Abstract

The purification of virus particles and viral vectors for vaccine and gene therapy applications is gaining increasing importance in order to deliver a fast, efficient, and reliable production process. Ultrafiltration (UF) is a widely employed unit operation in bioprocessing and its use is present in several steps of the downstream purification train of biopharmaceuticals. However, to date few studies have thoroughly investigated the performance of several membrane materials and cut-offs for virus concentration/diafiltration. The present study aimed at developing a novel class of UF cassettes for virus concentration/diafiltration. A detailed study was conducted to evaluate the effects of (i) membrane materials, namely polyethersulfone (PES), regenerated cellulose (RC), and highly cross-linked RC (xRC), (ii) nominal cut-off, and (iii) UF device geometry at different production scales. The results indicate that the xRC cassettes with a cut-off of approximately 500 kDa are able to achieve a 10-fold concentration factor with 100% recovery of particles with a process time twice as fast as that of a commercially available hollow fiber. DNA and host cell protein clearances, as well as hydraulic permeability and fouling behavior, were also assessed.

## Introduction

Viruses and virus like particles (VLP) are playing an increasingly important role in the vaccine gene and cell therapy fields. Adenoviruses (Ads), in particular, are considered one of the most suitable platforms for production of viral vaccines and gene therapy vectors; they are medium-sized (90–100 nm), nonenveloped, icosahedral viruses composed of a nucleocapsid and linear, non-segmented double stranded (ds) DNA genome that is about 36 kb long. The use of recombinant Ads for vaccination and gene therapy requires fast and highly efficient purification protocols that yield high recovery of infectious particles, maintain viral infectivity, and effectively remove contaminating DNA and host cell proteins, while also concentrating the viral samples for final delivery.

The downstream purification train of biopharmaceuticals has been extensively developed in the past years by combining different chromatographic steps, namely ion-exchange [1] and size-exclusion chromatography (and, less frequently, affinity chromatography), intermingled with concentration and ultra/diafiltration steps [2]–[7].

Ultrafiltration (UF) is a key operation, as large-scale processes produce high volumes of bulk (up to 2 kL for vaccines or 20 kL for mAbs [8]) that must be concentrated 10–100 times to be further purified by chromatography. The volumetric concentration and buffer exchange of virus bulks are critical not only to obtain high titer vector stocks in the proper formulation buffer, but also to reduce the handled volume; the latter accelerates the downstream processing and keeps the scalability of the purification train at a manageable level [9].

UF membranes can be synthesized from different polymers, such as regenerated cellulose (RC), polysulfone (PS), polyethersulfone (PES), or polyvinylidene fluoride (PVF), although RC and highly cross-linked RC display better trade-off between low (unspecific) protein binding, mechanical strength, and resistance to cleaning procedures (chemical agents and temperature).

UF is usually operated in tangential flow mode, where the cross flow at the membrane surface creates a “sweeping action” that avoids or lessens concentration polarization and gel layer formation, thus inhibiting membrane clogging. UF processes are usually operated at constant transmembrane pressure,



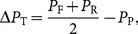
(1)


where 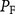 is the feed pressure, 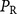 is the retentate pressure, and 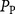 is the permeate pressure. However, constant permeate flux or constant permeate pressure operations are also implemented in practice [10]. These are normally preferred when unfavorable effects, such as enhanced fouling or product quality deterioration, are associated with high concentration of retained species at the membrane wall [11]. The work presented here is, however, focused on constant-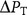 operation.

In viral downstream processes, 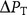 is usually between 0.5 and 1.4 bar, while the optimal cross flow rates can vary greatly due to the different structural stabilities of the various types of viruses; enveloped viruses are more labile than non-enveloped viruses and, thus, more prone to shear-induced damage [12], [13].

The membrane modules can also be assembled under different arrangements, for example flat sheet cassettes (FSCs) or hollow fibers (HFs). The majority of the published work refers to the use of HF modules for virus processing [14], [15] due to the fact that HF modules provide wider flow paths with lower shear rates [14], [16].

UF has been widely used both for concentration and for buffer exchange (diafiltration, DF), and is present in almost every virus DSP described in the literature [12], [13], [17]–[22] and disclosed patents [23]–[25]. The membranes used in virus UF have MWCOs in the range of 100–750 kDa allowing for high virus recovery (70–85%).

Despite the effort in developing robust downstream processes and platforms, most of the research in the field of virus purification has been focused on the chromatographic steps. Indeed, only a few works have investigated thoroughly the concentration/UF steps: Negrete et al. [26] optimized the use of a hollow fiber for concentration of virus like particles (VLP), while Wickramasinghe et al. [21] evaluated several PES membranes (micro and ultrafiltration) for the concentration of influenza virus; also, Grzenia et al. [27] evaluated four different small cut-off PES membranes for the purification of parvovirus particles. The rest of the literature is essentially focused on virus removal by UF membranes [28]–[30]. It should be pointed out that in the present work the viruses are not a contaminant but the product; therefore, the aim here is to concentrate and purify adenoviruses for viral vaccine or gene therapy applications.

The ideal UF membrane should have a very high separation factor, thus high product retention, and very high permeability. As correctly pointed out by Metha et al. [31] and Cramer et al. [32] such membranes are currently not available in the market.

The present study aimed to develop a novel class of UF cassettes for adenovirus type 5 (Ad5) concentration/diafiltration. Typical membranes with cut-offs in the range of 300 to 1000 kDa were compared, since most of the HF membranes for viral processes have these cut-off sizes. While PES membranes with pore sizes in this range are known to have quite open structures, RC membranes are typically much tighter even when their pore size is not narrowed by adsorptive phenomena during process operation; thus, current commercially available RC membranes are not suited for typical viral vaccine processes (virus diameters around 100 nm).

A detailed study was conducted to evaluate the effect of membrane material, namely PES, RC, and highly cross-linked RC (xRC), nominal cut-off, and ultrafiltration device geometry. In the first part of the work, the hydraulic permeabilities of a set of membranes, which include eight R&D prototypes were evaluated. Hydraulic permeabilities were assessed for the clean membranes, after usage, and after cleaning-in-place (CIP). In the second part of the work, a 10-fold concentration step followed by 5 diafiltrations was performed under constant-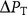 conditions. The separation factor (total Ad5 recovery and infectivity) and selectivity towards the major impurities, such as host cell proteins (HCPs) and DNA, were determined and the throughput of each device was appraised. The outperforming membranes were then scaled up and produced in standard manufacturing equipment. Ultimately, the present study identified a new large cut-off membrane able to achieve a ten-fold concentration factor with higher throughput and 100% infective Ad5 recovery.

## Materials and Methods

### Adenovirus production

The Ad5 production based on HEK 293 cells cultured in Ex-cell 293 serum-free media was performed in a 5 L working volume bioreactor (Sartorius Stedim Biotech, Germany). The dissolved oxygen was controlled at 50% air saturation by a 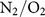/air mixture delivered by a sparger. The aeration rate was 0.01 vvm (vessel volumes per minute). The pH-value was controlled at 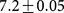 by aeration with 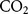 in the gas mixture and by base addition (1 M 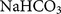). The temperature was controlled at 37°C using an external water-filled jacket. Mixing was provided by two 6-segment Ruston impellers with the agitation rate controlled between 60 and 210 rpm. The bioreactor inoculum was 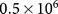 cells/mL, the cell concentration at infection (CCI) was 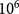 cells/mL, and a MOI of 5 was used. The bioreactor was harvested 48 hours post infection (hpi).

### Harvest and clarification

After the Ad5 bioreactor harvest, the cells were lysed by adding Triton X-100 (X100, SIGMA-ALDRICH, Switzerland) to a final concentration of 0.1% (w/w). Simultaneously, Benzonase (Merck Millipore, Germany) was added to a final concentration of 50 U/mL. The virus-containing bioreactor bulk was incubated at 37°C for 2 h.

Clarification of the Ad5 bulk was performed using a Sartopore 2 filter with 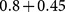 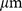 pore size (Sartorius Stedim Biotech, Germany). Before filtration, the module was primed with 3 capsule volumes of TRIS buffered saline, pH 8.0 (Sigma-Aldrich, Switzerland). The virus-containing bulk was loaded to the filter at a constant flow rate equivalent to 150 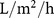 (LMH) using a Tandem 1082 Pump (Sartorius Stedim Biotech, Germany). The 5 L clarified bulk was transferred into 450 ml Nalgene bottles and frozen at −80°C until further use. The performances of the UF membranes were all assessed with material from this batch with the same concentration of Triton and Benzonase in order to avoid any batch-to-batch variability.

### Ultrafiltration setup

**Figure 1:**
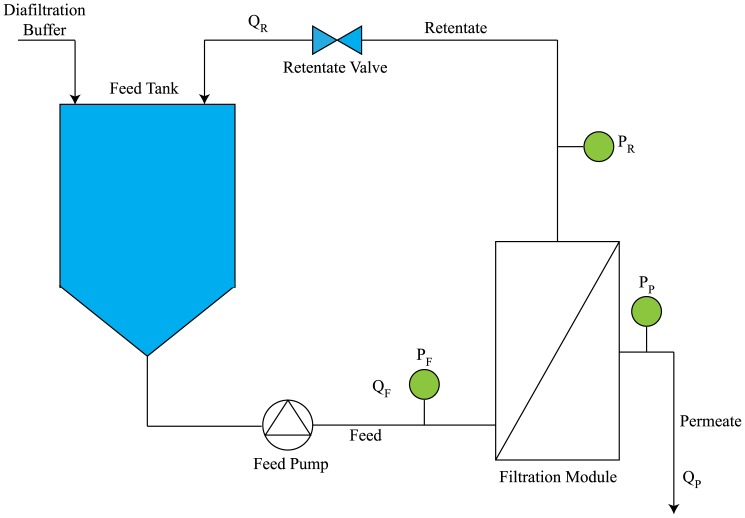
Set-up of the filtration unit employed in the experiments. The bulk was fed using a Tandem 1082 Pump and the retentate recirculated to the feed tank. the pressure was monitored by in-line transducers at the inlet, retentate outlet, and permeate outlet. 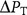 was kept constant at 1–1.2 bar by a flow restriction valve. TRIS saline buffer at pH 8 was used as diafiltration buffer.

The ultrafiltration cassettes were kindly provided by Sartorius Stedim Biotech, Germany, while the 750 kDa hollow fiber module was purchased (GE Healthcare Life Sciences, Sweden). The membrane modules were set up according to the manufactures' instructions. Briefly, a Tandem 1082 Pump (Sartorius Stedim Biotech, Germany) was used to pump the clarified bulk into the membrane device, the retentate was recycled to the feed container, and the permeate was collected separately (Fig. 1). The transmembrane pressure, 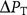, was controlled by adjusting the retentate flow rate using a flow restriction valve.

Before the experiments, the membranes were thoroughly rinsed with ultrapure water (Grade 1, as defined in ISO 3696). The membrane permeability was determined by the normalized water permeability at 20°C, 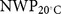, expressed in units of 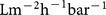 (LMH/bar).

The 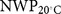 was calculated based on pure water permeate fluxes (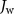) measured at five different values of 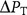 between 0.4 and 2.0 bar, according to 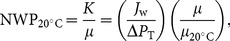
(2)where the viscosity ratio 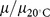 is a temperature correction factor that adjusts the value of NWP from the experimental temperature to 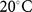 [33]. The 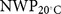 value was measured before and after each experiment, and after the CIP.

After conditioning the UF system with diafiltration buffer (20 mM TRIS, pH 8.0, 25 mM NaCl), 450 mL of clarified feedstock containing Ad5 were concentrated 10-fold and then diafiltered five times. The UF/DF test was performed at a constant 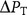 of 1.2 bar and at a constant feed flow rate (cross-flow) equivalent to a linear velocity 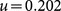 m/s (cf. Table 1). The linear velocity was the same in all experiments to ensure the same tangential flow force in the various prototypes in order to properly assess their performance with respect to MWCO and type of membrane material. As shown in previous work by our group and others [20], [26], [34], a 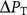 of 1.2 bar is a suitable pressure value for virus concentration.

**Table 1:** Membrane characteristics and feed flow rates used.

Membrane	Development stage	Filter material	Filter area	Configuration	Dimension	Cross section	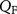
			(cm 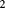 )		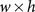 ( 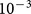 m)	( 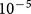 m 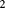 )	(mL/min)
Type B 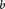	R&D prototype	RC	200	Cassette	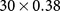	1.14	138
Type C 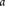	R&D prototype	RC	200	Cassette	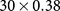	1.14	138
Type D 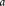	R&D prototype	xRC	200	Cassette	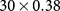	1.14	138
Type E 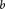	R&D prototype	xRC	200	Cassette	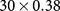	1.14	138
Type # 2 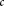	R&D prototype	PES	200	Cassette	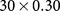	0.90	109
Type # 4 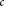	R&D prototype	PES	200	Cassette	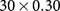	0.90	109
HF 3 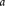	R&D prototype	PES	155	Hollow fiber	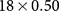 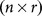	1.41	171
HF 5 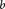	R&D prototype	PES	155	Hollow fiber	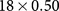 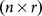	1.41	171
Type F 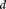	Pilot production	xRC	200	Cassette	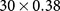	1.14	138
Type H 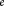	Pilot production	xRC	200	Cassette	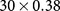	1.14	138
HF 7 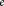	Commercial	PES	225	Hollow fiber	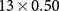 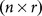	1.02	124

RC: regenerated cellulose.

xRC: regenerated cellulose modified with a highly hydrophilic cross-linking.

PES: polyethersulfone.

*a*, *b*, and *c* are, respectively, the low, medium, and high MWCO prototypes within the 300–1000 kDa range.

*d* and *e* are, respectively, the low and high MWCO prototypes within the 500–750 kDa range;

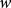
 and 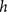 are the width and height of the cassette flow channel.

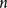
 is the number of fibers in the hollow fiber module and 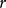 is the internal radius of the fibers.

The membrane load was kept constant at 22.5 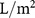 for the membranes with areas of 200 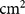 and 225 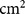, whereas for the devices with 155 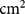 the membrane load was 29.0 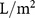. This difference was due to the minimum working volume of 45 mL allowed by our experimental set-up. Throughout the filtration process, samples of the retentate (1 mL) were collected and stored at 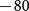
°C for further analysis.

The shear rate at the wall, 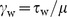, where 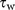 is the shear stress and 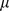 is the fluid viscosity, was calculated as follows for the two membrane configurations [35]: 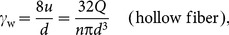
(3)




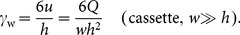
(4)Here, 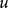 and 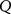 are the cross-flow mean fluid velocity and volumetric flow rate, respectively; 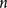 is the number of fibers of the hollow fiber, and 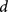 is inner diameter of the fibers; 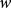 and 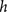 are the width and height of the cassette channel.

The CIP procedure consisted of washing the UF system with 1 M NaOH at a flow rate of 500 mL/min and then a 60-min incubation. After this treatment the system was rinsed with ultrapure water until the outlet stream reached pH 7. For the Ad5 runs, all procedures were performed at 20–22°C.

### Scanning Electron Microscopy (SEM)

SEM images of membrane cross sections were prepared by rinsing the membrane samples in Arium pure water and soaking them with Sakura Tissue Tek O.C.T compound resin prior cutting the membranes with a Leica CM3050 S cryo microtome. The samples were sputtered with a 5 nm layer of gold under vacuum in an Emitech K550 Sputter Coater and subsequently transferred to the FEI Quanta 200F SEM featuring a FEG (Schottky field emission gun) and ETD (Everhart Thomley Detector) under high vacuum (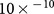 mbar) for imaging. Version 2.4 of xT Microscope Control software was used for image collection and instrument control.

### Sieving curve

A mixture of technical dextrans (purchased from SERVA Electrophoresis) was prepared in pure water added with 0,05% sodium azide (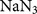). A sample of this feed solution was prepared for SEC analysis. Filtration was performed for flat sheet membranes in Amicon stirred cells (type 8200) under quasi non convective flow (TMP ¡ 20 mbar) or for hollow fiber modules using a peristaltic pump under similar conditions. Samples were collected at the permeate outlet after equilibrating the membrane and discarding twice the dead volume on the permeate side. Retentate samples were taken after filtration. A subsequent SEC analysis was performed on an Agilent 1100 integrated SEC system using a PSS Suprema Linear XL column. Pure water with a content of 0.05% sodium azide (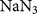) was used as an eluent at a flow rate of 1 mL/min. Detection of the polymer was performed via RI detection. A dextran standard calibration based on narrowly distributed PSS polymer standards was used to determine the proper molecular weight. Molecular weight distribution of feed, permeate and retentate samples as well as sieve curves and cut-offs were calculated using PSS Unichrom software.

### Gold nanoparticles rejection protocol

Gold nanoparticle solutions with particle sizes of 50 nm and 100 nm were purchased from BBI, sodiumdoceylsulfate (SDS) was purchased from Sigma-Aldrich, and pure water was produced using an Arium pro VF Ultrapure Water System. An amount of 1 g of SDS was dissolved in 1000 g of pure water under vigorous stirring. Afterwards, 10 mL of each solution of gold nanoparticles (50 nm and 100 nm) were diluted with 90 mL of SDS solution (1 g/L). The diluted solutions were stored in the fridge at 3°C and equilibrated under room temperature prior to filtration.

Filtration was performed in an Amicon 8010 stirred cell. First, 10 mL of SDS buffer were filtrated by applying a pressure of 1 bar. Subsequently, 10 mL of the diluted solution were transferred into the Amicon stirred cell and 8 mL were filtered by applying a pressure of 1 bar while stirring at 1100 rpm. Extinction of feed solution permeate was determined by UV-/vis-spectroscopy using a PerkinElmer Spectrophotometer Lambda 16 at a wavelength of 524 nm (50 nm nanoparticles) and 570 nm (100 nm nanoparticles).

### Total virus particle quantification

Total virus particle concentration and size distribution were measured using a NanoSIGHT NS500 (NanoSIGHT Ltd, UK). The samples were diluted in D-PBS (Gibco, UK) to get a virus concentration in the instrument's linear range (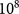
–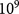 particles/mL). For each sample, three 60-second videos were acquired and particles between 70 and 130 nm were considered.

### Infectious virus particles titration

For Ad5 titration, 293 cells were seeded at 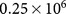 cells per well in 24-well flat bottom plates (Nunc, Denmark). After 24 h, the cells from three wells were trypsinized and the cell concentration was determined. The cell culture medium was removed from the remaining wells and replaced with 1 mL of viral suspensions (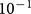
–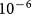) diluted in fresh medium. After 17 to 20 hours, the cells were collected in Dulbecco's phosphate-buffered saline (D-PBS, Gibco, UK) with 5% FBS and immediately analyzed by flow cytometry (CyFlow space, Partec GmbH, Germany). Both the initial feedstock and the samples collected during the 10-fold concentration steps were analyzed in the same assay, thus using the same cell culture, to eliminate assay-to-assay variability. The infectious particle (IP) recovery, 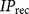, was calculated as follows:



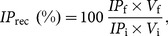
(5)


where 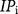 is the initial infectious particle concentration and 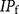 is the value at the end of the 10-fold concentration step; 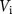 represents the initial volume and 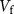 the final volume obtained after the 10-fold concentration step.

### DNA quantification

Total DNA was quantified using the fluorescence-based Quant-iT PicoGreen assay kit (Invitrogen, UK) according to the manufacturer's protocol. In order to avoid matrix interference, the samples were diluted between 2–256-fold with the reaction buffer provided.

### Protein analysis

Total protein was quantified using the BCA Protein Assay Kit (Thermo Fisher Scientific, USA) according to the manufacturer's protocol. Bovine serum albumin (BSA) was used for the calibration curve. In order to avoid matrix interference, the samples were diluted between 2 and 256-fold.

Host cell protein was quantified using the HEK 293 HCP ELISA Kit (Cygnus Technologies, USA) following the manufacturer's protocol. The standard curve was done using the provided 293 HCP standards. Dilutions of 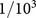, 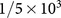, and 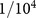 were performed in order to avoid dose hook effects and to allow interpolation. All analytical assays were performed in triplicate.

## Results and Discussion

The selection of an appropriate membrane to improve the concentration/diafiltration step is critical to ease the entire downstream train. With this objective in mind, three membrane materials—RC (regenerated cellulose), xRC (highly cross-linked RC), and PES (polyethersulfone)—were evaluated. First, the evaluation of the R&D prototype is presented; then, the selected best performing membrane is manufactured and scaled-up in a standard manufacturing casting line. The cut-offs of the R&D pilot and production prototypes ranked between 300 and 1000 kDa. Table 1 summarizes the membrane characteristics, which are discussed in detail below.

### Evaluation of R&D membrane prototype

#### Hydraulic permeability

**Figure 2:**
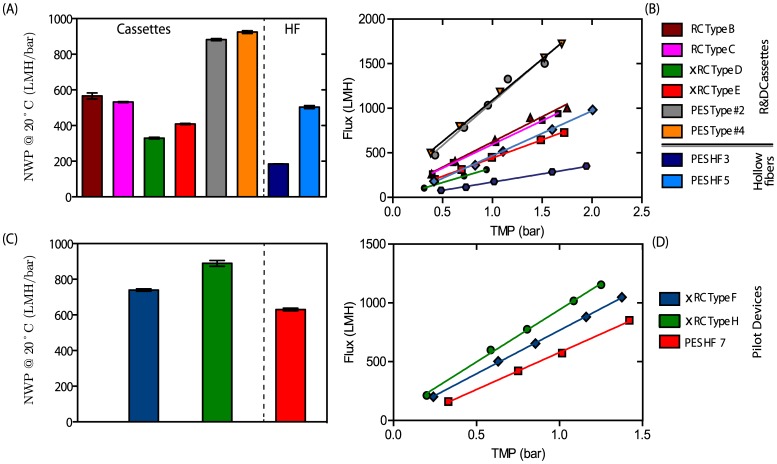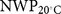 (average 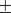 SEM) for the different UF membranes. (A) R&D prototype devices with different materials, namely RC, xRC, and PES. (C) The pilot production devices were only made of xRC and compared against commercially available GE HF 750 kDa (PES UF 7) modules. (B, D) water flux (LMH) at various values of 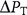 ranging between 0.5 and 2 bar.

All eight UF prototypes were characterized by their normalized water permeability, 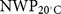; these values are plotted in Figs. 2A and 2B. As expected, the results show that increasing the pore size leads to an increase in permeate flux for the same transmembrane pressure (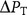). This is in agreement with the findings of Wickramasinghe et al. [21] and other authors.

The two PES cassettes (type #2 and type #4) registered the highest 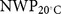, ca. 880–925 LMH/bar, and MWCO around 1000 kDa. Although both PES cassettes are within the same MWCO range, the PES type #4 cassette was designed to be slightly more open; this is confirmed by its enhanced 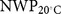. Both RC cassettes exhibited 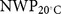 values close to 560 LMH/bar, whereas that of xRC only reached 400 LMH/bar. The HF modules showed lower permeabilities than the PES and RC-based cassettes. As expected, the lower MWCO of PES HF 3 (roughly 300 kDa) resulted in lower permeability than PES HF 5 (roughly 500 kDa).

The water flux permeability was measured after membrane usage and after cleaning in place. This measurement assesses the degree of irreversible/strongly associated foulants accumulated on the membrane during filtration. On the other hand, the after-CIP flux recovery gives the loss of permeability after a complete cycle. This metric is an industrially relevant indicator as it serves as benchmark of membrane performance, and is used to assess performance decay as well as the effectiveness of CIP protocols.

**Figure 3:**
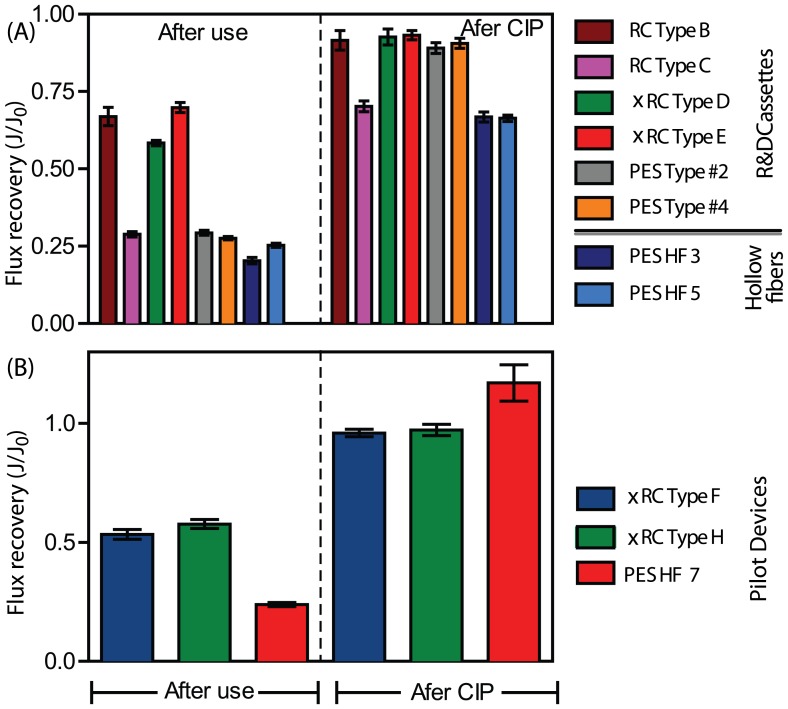
Flux recoveries (average 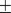 SEM) after usage and after CIP with 1 M NaOH for (A) different R&D prototypes and (B) pilot production devices. In both cases the highly cross-linked regenerate cellulose (xRC) was able to achieve higher flux recovery after usage than the PES-based membranes. After CIP, all membranes recovered their initial flux except for the PES HF 3 and 5 modules and RC type C membrane.

The flux recoveries, 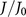, where 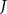 and 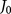 are the fluxes after and before membrane usage, are given in Fig. 3A. The water flux recovery after use was nearly 3-fold greater for the RC and xRC than for the PES-based material. A significant water flux permeability decay indicates the presence of fouling or nonspecific, irreversible adsorption. Since PES is hydrophobic, this leads to increased fouling compared to the regenerated cellulose.

However, except for the PES-based HF prototype and RC Type C, after CIP with NaOH 1M the flux was restored in all cassettes. The type B membrane and the two xRC cassettes regained their permeability, since their flux loss ranged between 7% and 11%, indicating that these UF modules withstood a complete cycle and might be used as a repeated-use device; the effect of the number of cycles on the long-term behavior of these membranes was not assessed. The remaining modules (RC type C and the two PES hollow fibers) showed a decreased of water permeability after one cycle; these devices are not suited for repeated use.

#### Virus Recovery

**Figure 4:**
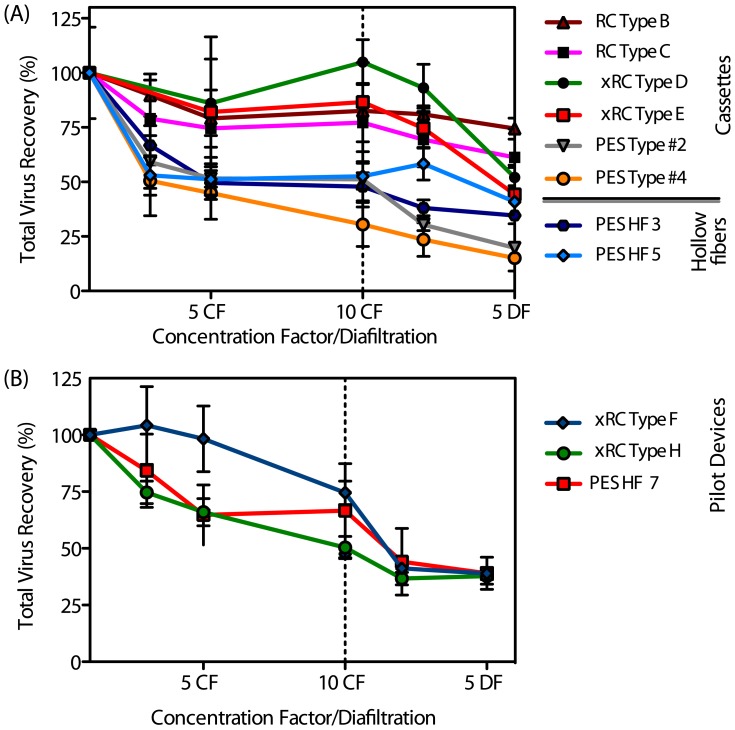
Total virus particle recovery as a function (average 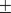 SEM) of the concentration diafiltration volume for (A) different R&D prototypes (B) pilot production devices. The plots display the same process first operating in concentration mode and then diafiltration. In both cases the highly cross-linked regenerate cellulose xRC showed the highest recovery yield when compared to PES menbranes.

Ultrafiltrated samples were collected at the retentate side upon achieving 3, 5, and 10 concentration factors (CF) and also after 2 and 5 diafiltrations (DF). The samples were further analyzed to determine the filtration performance of each UF device. Fig. 4A and Table 2 show the total particle and infectious particle recoveries, respectively.

**Table 2:** Recovery of infectious particles (IP) and processing time for 10-fold concentration for the different pilot production and R&D UF devices.

Device	Material	Water flux	Processing	IP	DNA	HCP	Shear
			time	recovery	clearance	clearance	rate
		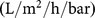	(min)	(% 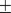 SEM)	(% 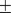 SEM)	(% 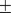 SEM)	( 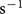 )
Type B	RC	590	14	94 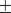 14	57 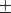 0.76	68 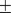 3.91	3186
Type C	RC	566	30	85 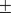 11	55 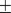 0.63	73 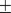 6.54	3186
Type D	xRC	390	24	72 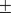 11	63 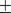 1.71	79 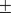 4.72	3186
Type E	xRC	400	14	100 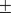 13	57 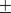 3.91	71 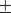 9.78	3186
Type #2	PES	900	19	89 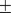 16	70 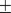 2.71	86 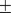 8.67	4037
Type #4	PES	924	17	54 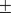 11	73 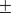 4.27	90 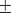 2.8	4037
HF 3	PES	185	105	60 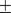 4	64 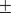 2.59	77 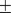 12	1613
HF 5	PES	504	48	8 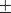 2	51 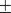 2.41	70 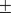 0.72	1613
Type F	xRC	739	17	100 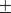 12	48 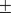 0.90	57 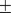 20.1	3186
Type H	xRC	889	17	93.6 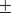 16	46 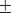 0.61	58 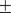 9.64	3186
HF 7	PES	630	26	100 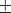 13	66 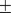 1.78	86 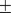 22.9	1619

All RC-based cassettes achieved total particle recoveries ranging from 69% to 93% after 2 DFs. Contrarily, the PES-based membranes permeated virus particles, as indicated by the low total particle recoveries (23–58%). This was partially anticipated, since the PES cassettes presented the highest MWCO (ca. 1000 kDa).

The infectious particle (IP) data given in Table 2 are consistent with the total particle recoveries except for the PES Type #2 cassette. In particular, all the RC-based cassettes were able to recover between 79% and 100% of IPs at the end of the concentration step. Interestingly, of these four modules, the ones with larger MWCO (RC type B and xRC type E) were those yielding higher IP recoveries, close to 100%. This result points out the importance of optimizing the choice of cassette and MWCO for the lowest processing time to avoid loss of infectivity.

Following the trend observed for total particle recovery, the type #2 PES cassette recovered more infectious particles than the PES #4 module. Noteworthy, is the higher recovery of infectious particles than that of total particles; this is more pronounced for the PES type #2 prototype. The reason for this can be attributed to the presence of incomplete viral particles or empty capsids that are removed during the ultrafiltration process by several mechanisms, such as adsorption and sieving and/or entrapment in the larger pores. Vellekamp et al. [36] showed that empty capsids have a slightly different shape than infectious particles, and their surface appears rounder when observed by electron microscopy. Their stability might also be compromised and, therefore, they may be are more prone to shear stress damage. These features, combined with the higher permeability of the PES Type #2 cassette, may explain the enhanced infectious viral recovery and reduced total particle recovery. Also, the PES Type #2 cassette performed poorly in the flux recovery test after usage, suggesting the occurrence of virus entrapment leading to membrane fouling. The PES cassettes have the same type of membrane material and the same MWCO; however, it is known that slight differences, such as a broader (or narrower) pore size distribution, can impact the virus recovery achieved by the membrane [16]. Indeed, the PES#4 prototype was manufactured with a slightly broader pore size distribution; this is also evident by looking at the superior clean water flux that this membrane displays.

The HF prototypes yielded low IP recoveries. In particular, the HF 3 module took seven times longer than the type B or type E prototypes to complete the same 10-fold concentration step. Still, the HF 3 module showed a moderate IP recovery (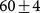
%). This can be explained by the longer processing time, which may give rise to enhanced adsorptive interactions between the hydrophobic PES material and the virus particles. The lower flux recovery after usage and after cleaning in place supports the hypothesis of strong hydrophobic-driven adsorption.

#### Impurity clearance

**Figure 5:**
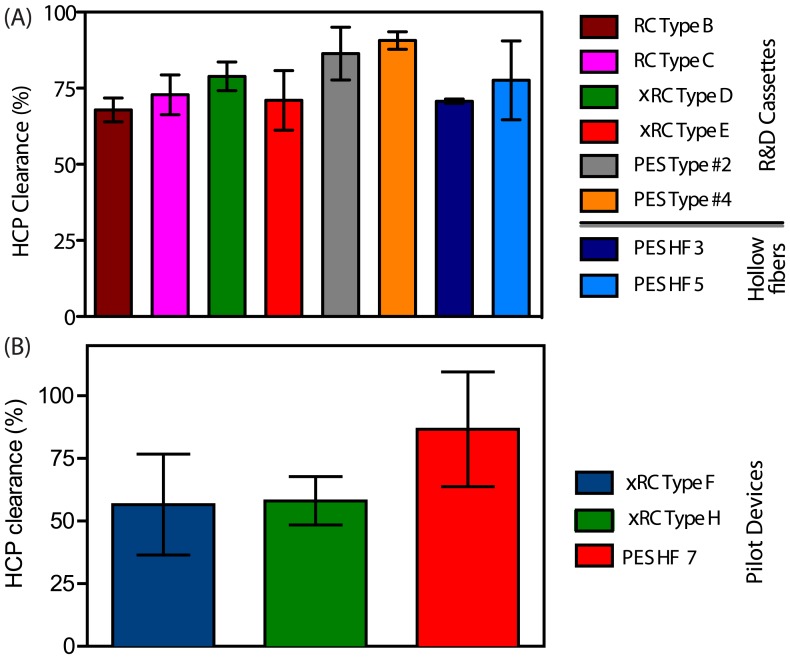
HCP clearance (average 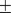 SEM) as a function of the concentration/diafiltration volume for different R&D prototypes (Fig.A) and for the pilot production devices (Fig.B). Both figure display the HCP clearance value after 10 fold concentration. The HCP clearance does not show important differences among the different cassettes and or HF. A slight increase in clearance is observed for the PES based cassettes and for the GE HF 750 kDa (PES HF 7). (For interpretation of the references to colour in this figure legend, the reader is referred to the web version of this article)

The HCP analysis given in Fig. 5A shows that the two PES cassettes were able to remove 86–91% of the HCP present; this was expected given their high MWCO and hydrophobic properties. The only exceptions are the HF 3 and HF 5 prototypes as these were in the 300 kDa and 500 kDa ranges, respectively. It is likely that high molecular weight HCPs can easily pass through the 1000 kDa pores of the PES membrane cassette.

**Figure 6:**
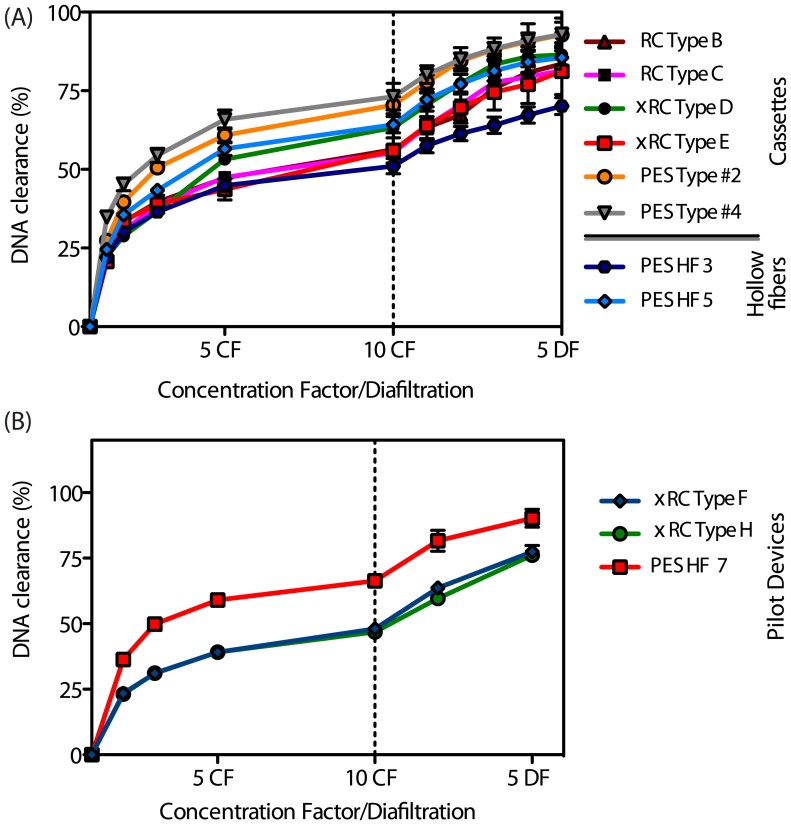
DNA clearance as a function (average 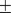
SEM) of the concentration/diafiltration volume for different R&D prototypes (Fig.A) and for the pilot production devices (Fig.B). The plots display the same process first operating in concentration mode and then diafiltration. At both R&D and pilot production scales, the PES-based membranes lead to an increased DNA removal compared to their RC and xRC counterparts. (For interpretation of references to color in this caption, the reader is referred to the web version of the article).

One of the challenges of Ad purification is host cell DNA removal, not only because the bioprocess comprises a cell lysis step [37] but also because it has been shown that DNA can associate with the virus particles resulting in co-purification of both species [3]. Both PES-based cassettes enabled higher DNA clearance, ca. 85%, than the remaining prototypes (Fig. 6). The types B, C, D, E, and HF 5 prototypes showed intermediate DNA clearances (67–70%) and the HF 3 showed the worst DNA clearance (61%) (Fig. 6 A). Both the HCP and DNA clearance data are in agreement and point out both PES cassettes as the best modules with respect to impurity removal. However, when the TP and IP data are also taken into account, these modules were not as efficient as the RC-based membranes.

#### Productivity

The permeate flux over time and membrane throughput (feed processed per membrane area per unit time, 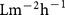, or LMH) were evaluated for all UF prototypes studied. The membranes with higher cut-off, namely type B and PES type #4, showed an initial marked flux decrease before a steady state was achieved. The higher the clean water flux, the lower is the resistance to the permeate side of the membrane. Low membrane resistance results in initially high permeate flow for the clean membrane. This will cause rapid convection of particles and small contaminants towards the membrane surface leading to rapid flux decline [21], [38].

**Figure 7:**
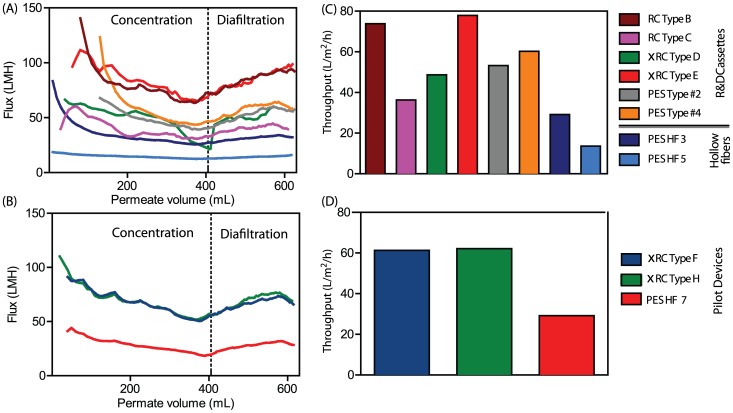
Flux decay curves as a function of the filtration time Fig.(A and (B). Throughput (liter of feed processed in the unit of time (h), given a defined membrane area (m^2^)) Fig. C and D. The value reported in Fig. C and D are after to 10-fold concentration and 2 diafiltration volume. For the large cut off R&D membranes a strong flux decay is observed at the beginning of the filtration(Fig A). Pilot production devices and GE HF 750 (PES HF 7) show the same decay profile (Fig.B). Cassettes with RC Type B and xRC Type E show the highest throughput (Fig. C), the throughput for the GE HF 750 is 2 fold less compared to the pilot production cassette membranes (Fig.D). (For interpretation of the references to colour in this figure legend, the reader is referred to the web version of this article)

Fig. 7A shows that all R&D membranes were able to maintain a stable flux due to the constant-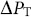 operation, except for type D which showed a marked flux decrease between 300 to 405 mL of permeate volume (between 3 to 10 CF). This indicates the starting point of fouling, which may be due to the low MWCO. Indeed, this membrane yielded the lowest clean water flux (Fig. 2).

### Identification of a suitable membrane

Among the R&D prototypes, the PES hollow fiber modules showed the worst performance under the conditions evaluated. This is supported by the lower IP recoveries and longer processing times. While the HF 5 module lost nearly all viruses, the HF 3 prototype was still able to recover 60% of infective particles but the long processing time is a considerable disadvantage. The results obtained with the PES R&D prototypes, namely the values of total particle recovery, are below what has been reported in the literature for this MWCO range. For instance, 300 kDa HFs from GE are able to concentrate 10-fold an Ad5 bulk with 90% IP yield [6]. The only exception is the PES Type#,2 which yielded an IP recovery of 89%. However, the lower flux recovery after use and after CIP makes this membrane less attractive for processing large volumes of feedstock.

The RC-based R&D prototypes showed better performances, especially with respect to the IP recovery. In particular, type B and type E membranes registered superior IP recoveries than what has been reported for Ad5 ultrafiltration [6], [13], [20], indicating that the shear rates used did not damage the adenovirus virions. These membranes displayed similar HCP clearance (68% and 71%) and DNA clearance (67% and 70%, respectively). The throughput capacities of these two modules are rather high, being able to process up to 77 L of feed in 1 hour using a 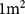 ultrafiltration module. It should be pointed out that the throughput analysis was done by taking into account a 10-fold concentration step and 2 buffer exchanges. Considering that UF is often employed at an early stage in the downstream processing train, high recovery yields and high throughput capacities are preferred over enhanced impurity removal rates, although here there are still remarkable.

**Figure 8:**
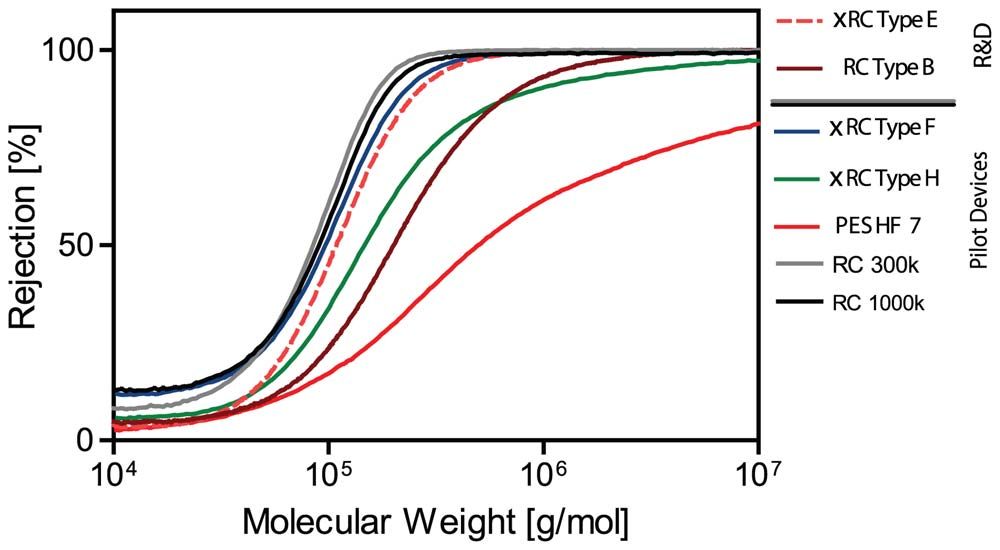
Sieving curve for visualization of different pore size by using different molecular weight dextrans, ranking between 10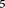 and 10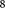 g/mol. RC and xRC exhibit a narrower rejection range than the other membranes and, therefore, a more homogeneous pore size distribution. Two commercially available RC cassette are compared against showing tighter pore sizes even for the membrane rated as 1000 kDa. GE HF 750 kDa (PES HF 7) exhibits the largest cut-off and wider pore size distribution. (For interpretation of the references to colour in this figure legend, the reader is referred to the web version of this article) PES based cassettes and HF both outperform the xRC and RC based cassettes.

**Figure 9:**
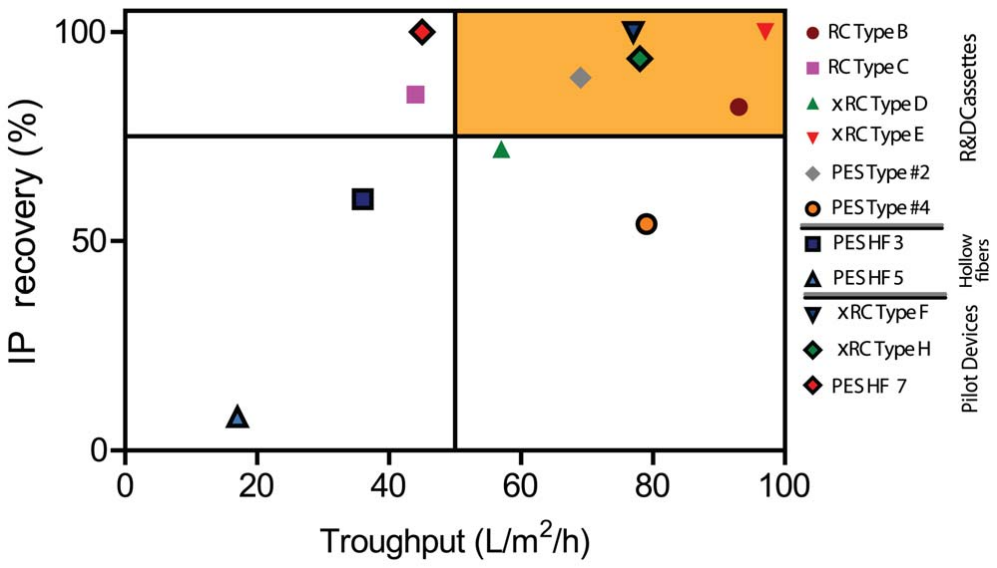
Trade-off between throughput, indicated as liter of feed processed in the unit of time (h), given a defined membrane area (m^2^), and infective particle recovery yield. The values referrer at 10 times concentration factor. The orange area on the right top corner depicts the best membranes. RC and xRC membranes showed the highest throughput coupled with high recovery yield. (For interpretation of the references to colour in this figure legend, the reader is referred to the web version of this article)

Taking into account these features, the type B and type E membranes served as base for the design and scale-up to the manufacturing casting line. Sieving curves showed that rejection profile of the selected R%D prototype is comparable to the profile of the pilot devices, namely type F and type H (Fig. 8). It is also important to point out that the two UF modules that displayed superior throughputs (Fig. 7 B), RC Type B and xRC Type E, were also the ones that gave the highest virus recoveries (Fig. 9). A commercial PES hollow fiber with 750 kDA MWCO from GE healthcare, hereafter referred to as PES HF 7, was chosen as benchmark reference. These new pilot production devices were evaluated by performing the same type of experiments for the R&D prototypes.

### Evaluation of the pilot production devices

The pilot-production cassettes were optimized to increase their water flux permeability. The membranes and cassettes were manufactured on standard production-scale manufacturing equipment and were designed to be scalable to large-area devices. xRC was the material chosen for the pilot production due to the goods values obtained for the various performance metrics discussed in this work. Relevant process performance, such as recovery of infectious particle (IP) and total particle (TP) recoveries, were the main drivers for this choice.

The xRC type H cassette (ca. 750 kDa) exhibited the highest 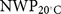 of the pilot devices, 889 LMH/bar, as seen in Fig. 2C and D. This is in accordance with its high MWCO as observed by the sieving curves plotted in Fig. 8. The type F cassette showed a smaller 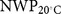 of 739 LMH/bar, but still larger than that of HF 7, which was determined to be 630 LMH/bar. The lower permeability of the GE 750 kDa HF (PES HF 7) is somewhat puzzling. The sieving curve in Fig. 8 shows a higher MWCO for HF 7 than for the cassettes, therefore a higher water flux would be expected. On the other hand, HF 7 has a much broader sieving curve than the cassettes, which indicates that the HF 7's permeability is controlled by the fraction of smallest pores in its pore size distribution.

Regarding the water flux after cleaning in place, the two xRC cassettes fully recovered their permeability (96% and 97% flux recovery). This provides a good indication being reusable. Indeed these membranes were also optimized to sustain several CIP cycles, as this is an important feature for process robustness. In particular, when comparing the Type B and E membranes water flux after CIP with the pilot production devices have an increased flux recovery of 3–4% (Fig. 3 A and B).

Contrarily, the HF 7 showed a performance decay after CIP, since its hydraulic permeability increased by 17%. This can cause a change in the product and/or impurities retention rate when the membrane is reused.

Concerning total particle recoveries ranged from 50% (type H) up to 75% (type F) after 10-fold concentration (Fig. 4 B); however, after two diafiltration (DF) volumes the recovery yield decreased to 40% for all tested membranes. This effect is essentially membrane independent and most likely due to the formulation of the diafiltration buffer; in particular increasing ionic strenght and/or adding polysorbate 80 might overcome this issues, as shown by Konz et al. [3]

As for the impurities clearance, xRC type F and type H cassettes removed 67 and 68% of HCP, respectively, while the PS-based HF 7 was able to achieve 86% HCP clearance (Fig. 5 B). The higher HCP protein clearance registered by the HF might be due to the different filter material, which is more hydrophobic than RC, and thus more prone to adsorption.

For the Type F and Type H pilot device cassettes, the DNA clearance after two DFs was 64 and 60%, respectively, while the HF 7 was able to remove 82% of DNA present in the Ad5 feed (Fig 6 B).

The superior performance in DNA clearance of the GE HF can be attribute to the higher MWCO as shown in Fig. 8 allowing DNA to go trough the permeate side. However, DNA can also adsorb onto the membrane surface due to the hydrophobic nature of the PES material.

Regarding the removal of impurities, the PES HF gave rise to a slightly improved DNA and HCP clearance comparing with xRC cassettes. This increased impurity removal might be due to adsorption phenomenon rather than a size exclusion mechanism as the filter material's proprieties are more hydrophobic [10], [39].

**Figure 10:**
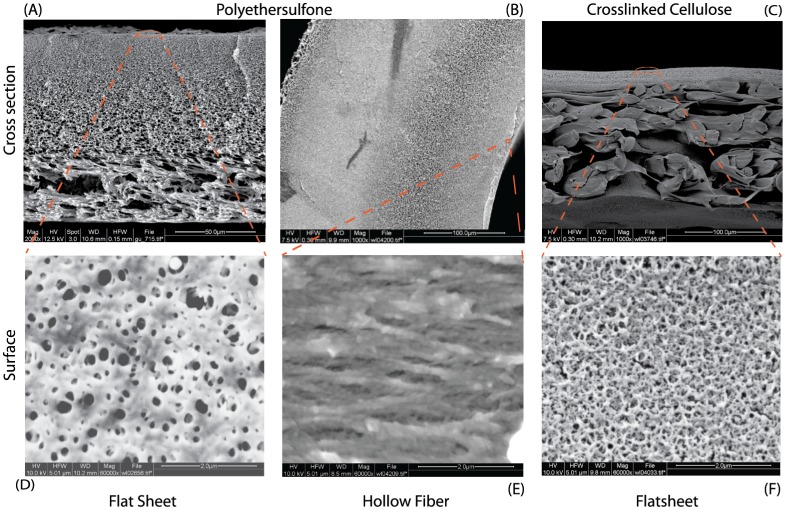
SEM pictures of PES membrane, xRC Type F membrane and HF 7. A, B and C indicate the cross section structure for the flat sheet membrane and hollow fiber, while D, E and F illustrate the surface structure.

Infectivity for the type F and HF 7 membranes was maintained yielding 100% of IPs, while Type H membrane achieved 94% IP recovery. Despite being rated with the same MWCO as the type H cassette, the HF 7 was able to recover a slightly more infective Ad5. The membranes were also evaluated based on their rejection of gold nanoparticles (GNP) of 100 nm (approximately the size of adenovirus) and 50 nm. Almost all the membranes showed a 50% rejection of the 50 nm GNPs and almost completely held back the 100 nm GNPs (Table 3). The membrane materials, xRC and PES, were also investigated by SEM analysis (Fig. 10). The main morphological difference appears to be related to the support layer, which shows a more neat and smooth pattern in the xRC. While one membrane is supported by a non-woven fabric, both filters feature an asymmetric sponge like structure, which is relevant for membrane stability but has low to none effect in the filtration process. All retention is performed at the very top layer of the filters (skin layer). Surface images confirm the asymmetry structures. (Fig. 10 D, E and F). Nevertheless within that structure adsorption of contaminants can occur due to steric or hydrophobic interaction, the latter of which is more present for PES based membranes.

**Table 3:** Characterization of xRC, RC and PES ultrafiltration devices.

Module	Material	Prototype	Permeability	Rejection	Rejection	Rejection
			protein solution	Glb (160 kDa)	GNP (50 nm)	GNP (100 nm)
			(LMH)	(%)	(%)	(%)
Cassette	RC	Type B	919	11	47	95
Cassette	xRC	Type E	179	47	52	96
Cassette	xRC	Type F	489	N/A	49	97
Cassette	xRC	Type H	1915	3	57	97
Cassette	PES	Type #2	83	81	43	96
Hollow fiber	PES	HF 7	130	32	63	95

Permeability of a protein solution of gamma-globuline (Glb) and rejection of gold nanoparticles (GNP) is shown.

The Type F cassette presented the best overall results among the assayed membranes. This is supported by the complete recovery of infective Ad5, with a remarkable improvement compared with the data described in the literature for 500 kDa HF [6].

Another important feature of the Type F (and H) cassette(s) is their higher throughput compared with the HF 7. For instance, the Type F module is capable of processing up to 61 L of Ad5 clarified bulk within 1 h using a 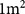-membrane while the GE HF can process only 29 L with the same time and membrane area (considering a 10-fold concentration and 2 DF).

Although the Type F throughput is 20% lower compared to those obtained for the best R&D prototypes previously evaluated, careful comparison of such values is required since the previous membranes' MWCO might be slightly different and the manufacturing process was different.

## Conclusions

This work presented an in-depth characterization of several ultrafiltration membranes. We have identified UF membrane modules alternative to the currently available HF devices traditionally used for virus purification. A cassette based on highly cross-linked regenerated Cellulose (xRC, close to 500 kDa and herein referred to as type E), which showed the best performance among the R&D prototypes, was successfully scaled up to a pilot production casting line. The resulting type F module showed better performance than a commercially available hollow fiber (750 kDa). The key advantage of this UF module is the substantially shorter processing time coupled with complete infectious particle recovery (100%) and is, therefore, suggested for Ad5 concentration.
